# The Associations of Lipid Profiles With Cardiovascular Diseases and Death in a 10-Year Prospective Cohort Study

**DOI:** 10.3389/fcvm.2021.745539

**Published:** 2021-11-25

**Authors:** Jiayi Dong, Song Yang, Qian Zhuang, Junxiang Sun, Pengfei Wei, Xianghai Zhao, Yanchun Chen, Xiaotian Chen, Mengxia Li, Lai Wei, Changying Chen, Yao Fan, Chong Shen

**Affiliations:** ^1^Department of Epidemiology, School of Public Health, Nanjing Medical University, Nanjing, China; ^2^Department of Cardiology, Affiliated Yixing People's Hospital of Jiangsu University, People's Hospital of Yixing City, Yixing, China; ^3^Department of Clinical Epidemiology, Geriatric Hospital of Nanjing Medical University, Nanjing, China

**Keywords:** lipids, apolipoprotein, derived lipid indices, cardiovascular diseases, death, cohort study

## Abstract

**Background:** Dyslipidemia is one of the modifiable risk factors for cardiovascular diseases (CVD). Identifying subjects with lipid abnormality facilitates preventative interventions.

**Objectives:** To evaluate the effects of lipid indices on the risks of ischemic stroke (IS), coronary heart disease (CHD), CVD, all-cause death, and CVD death.

**Methods:** The cohort study of 4,128 subjects started in May 2009 and followed up to July 2020. Restricted cubic spline (RCS) regression analysis was used to explore the dose-response relationship between lipid indices with outcomes. Cox proportional hazard regression analysis was used to estimate the association with a hazard ratio (HR) and 95% CI.

**Results:** RCS analysis showed that there were significant linear associations of TG with IS, non-high-density lipoprotein cholesterol (HDL-C), apolipoprotein B (ApoB), and total cholesterol (TC)/HDL-C ratio with all-cause death, non-HDL-C and RC with CVD death, and significant non-linear associations of ApoB with IS and CVD, TC, LDL-C, ApoAI, and TC/HDL-C ratio with CHD, and TC with all-cause death (all *P* <0.1). Cox regression analysis revealed that subjects with TC <155 mg/dl (vs. 155–184 mg/dl), > 185 mg/dl (vs. 155–184 mg/dl), and ApoB <0.7 g/l (vs. ≥0.7 g/l) had higher risks of CHD (*P* < 0.05), the adjusted HRs (95% CIs) were 1.933 (1.248–2.993), 1.561 (1.077–2.261), and 1.502 (1.01–2.234), respectively. Subjects with ApoAI > 2.1 g/l (vs. 1.6–2.1 g/l) and TG <80 mg/dl (vs. 80–177 mg/dl) had higher risks of CVD and all-cause death (*P* < 0.05), the adjusted HRs (95% CIs) were 1.476 (1.031–2.115) and 1.234 (1.002–1.519), respectively.

**Conclusions:** Lower or higher levels of TC, higher level of ApoAI, and lower level of ApoB were associated with increased risks of CVD, and lower level of TG was associated with increased all-cause death. Maintaining optimal lipid levels would help to prevent CVD and reduce mortality.

## Introduction

Cardiovascular disease remains a major cause of premature mortality and rising health care costs across the world (1). In China, the prevalence rate of cardiovascular disease (CVD) increased significantly by 14.7% from 1990 to 2016 (2), which was likely to increase substantially in pace with population growth and aging. Among the major drivers of CVD, atherogenic dyslipidemia is one of the major and modifiable risk factors for CVD (3, 4). In recent years, the blood lipid level of the Chinese population has shown a significant upward trend (5), with a 30% and above prevalence rate of dyslipidemia in adults, whereas the rates of awareness, treatment, and control of dyslipidemia remain low (6). Therefore, it is necessary to evaluate the effect of lipid profiles on CVD and mortality, identify the high-risk population, and tailor risk reduction strategies.

Epidemiological studies have investigated the associations between lipid profiles and CVD, including stroke and coronary heart disease (CHD). Two large-scale, prospective cohort studies indicated that the elevated levels of conventional lipids indices including total cholesterol (TC), low-density lipoprotein cholesterol (LDL-C), and triglycerides (TG), and the reduced level of high-density lipoprotein cholesterol (HDL-C) contributed to the increased risk of CVD incidence (7, 8). However, there was an inconsistency of the relationships between TC, HDL-C, LDL-C, and the risk of CVD incidence of all-cause death with a “U” or “J” pattern (9, 10). Due to the limitation in using a single lipid index to evaluate the relationship between dyslipidemia and CVD events and all-cause mortality, some studies proposed that apolipoprotein B (ApoB), apolipoprotein AI (ApoAI), lipoprotein (a) [Lp(a)] or lipid ratios had an additional clinical value, and recommended to attach importance to the measurement of ApoB in clinical practice (11–14). Interestingly, some studies indicated that the apolipoproteins and calculated lipid ratio as derived lipid indices have comparable CVD predictive value with conventional lipid indices (15, 16).

Most of the previous studies dealt with lipid indices as continuous variables for linear correlation or divided lipid indices into categorical variables according to the existing guidelines and percentage scales. Limited data were available on the non-linear relationship between lipids, especially the effect of low blood lipid and CVD events and mortality. Recently, restricted cubic spline (RCS) analyses have been widely used to explore the dose-response relationship in public health research. Therefore, this prospective cohort study aimed to quantitatively evaluate the effects of lipid profiles on CVD and death with a 10-year follow-up.

## Methods

### Data Source and Study Population

**Figure 1:**
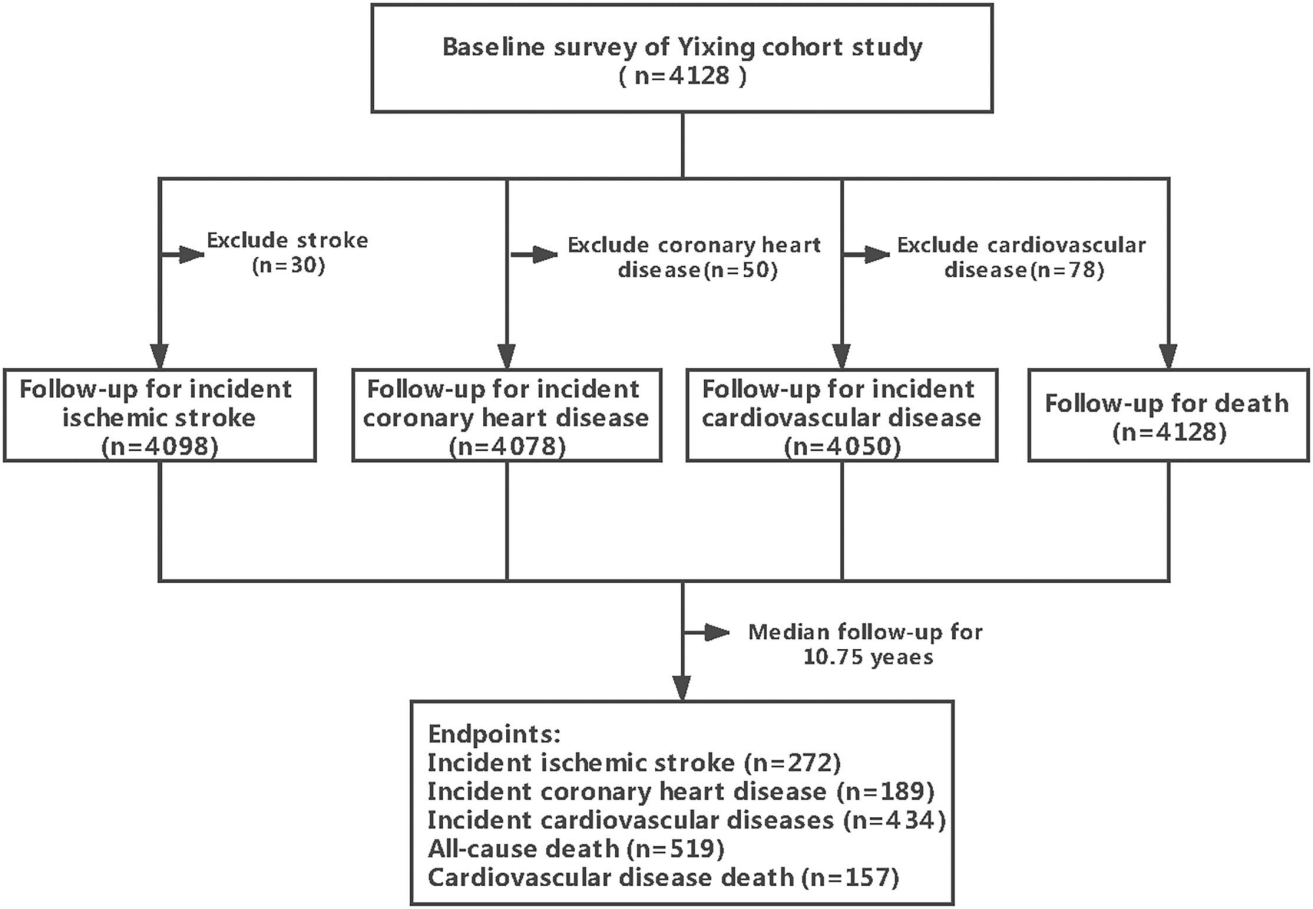
The flow chart of the cohort study.

A cluster sampling survey was conducted in Guanlin and Xushe towns at Yixing City, Jiangsu province, from May 2009 to August 2009 (17). A total of 4,128 community-dwelling adults aged 24–96 years old were included at baseline. The first field follow-up survey proceeded from May to October in 2014. From the local disease and death register system of the centers for disease control and prevention (CDCs), CVD and death events were further followed up till July 27, 2020. The flow chart of study subjects is shown in Figure 1. All the subjects were followed for the outcome of all-cause death (*n* = 4,128), while the subjects with the corresponding disease at baseline were excluded for IS patients (*n* = 30), coronary heart disease (CHD) patients (*n* = 50), and CVD patients (*n* = 78).

All subjects were well-informed and signed informed consent before inclusion in the cohort study. The study was approved by the ethics committee of Nanjing Medical University (#200803307), Nanjing, China.

### Data Collection and Definitions of Exposure

All subjects were interviewed using a questionnaire, underwent physical examinations and laboratory tests by trained investigators as previously described (18).

Smokers were defined as individuals who smoked ≥ 20 cigarettes/week lasting for at least 3 months a year. Drinkers were defined as those whose current or past alcohol consumption ≥ two times per week lasting for at least 6 months per year. Body mass index (BMI) was calculated as weight (kg)/height squared (m^2^). Individuals with an average systolic pressure (SBP) ≥ 140 mmHg or diastolic pressure (DBP) ≥ 90 mmHg, or a self-reported hypertension history or currently receiving antihypertensive medication were defined as hypertension cases. Individuals with fasting plasma glucose (FPG) ≥ 7 mmol/l, or a self-reported diabetes history, or currently receiving hypoglycemic medication were defined as diabetes cases.

### Lipid Indices Detection and Categorization

Blood samples were collected after overnight fasting (>8 h), plasma TC, TG, HDL-C, LDL-C, ApoAI, ApoB, Lp(a), and glucose (GLU) levels were measured with an automatic biochemistry analyzer Olympus AU2700. Non-high-density lipoprotein cholesterol (non-HDL-C), remnant-cholesterol (RC), TC/HDL-C, TG/HDL-C, LDL-C/HDL-C, and ApoB/ApoAI were further calculated. The non-HDL-C value was calculated as TC minus HDL-C. RC was calculated as TC minus LDL-C minus HDL-C. The judgment of abnormal lipid levels determined dyslipidemia according to commonly detected clinical lipid indices of TC, TG, HDL-C, and LDL-C (TC ≥ 240 mg/dl, or TG ≥ 200 mg/dl, or LDL-C ≥ 160 mg/dl, or HDL-C <40 mg/dl) or the self-reported history of the disease, or taking lipid-lowering drugs (19).

Lipid indices were categorized according to the dose-response relationships with CVD events and mortality tested by RCS regression analyses. If the relationship of lipid parameter and outcome was linear or no overall association, considering selecting the median or guidelines reference value as a cut-off point, if the median was close to the guidelines reference value, choose the latter as the final cut-off point, otherwise select the former. If the relationship of lipid parameter and outcome was U-shaped, select the lipid level with the corresponding HR of 1 in the RCS regression analyses or guidelines reference value as cut-off points. If there was a range with a corresponding HR value of 1, and the range was close to the guideline reference, the latter was chosen as the final cut-off point, otherwise the former was selected.

### Outcome Ascertainment

Outcome events of stroke, CHD, CVD, death, and CVD death were recorded from the disease and death register system, further inspected by certified neurologists and cardiologists of People's Hospital of Yixing City. International Classification of Diseases, Tenth Revision, Clinical Modification (ICD-10-CM) was used to identify for stroke (I60–I64), ischemic stroke (I63), hemorrhagic stroke (I60, I61, I62, and I64), and CHD (I20, I21, I22, I25.5, I25.6, and I46.1). In this study, CVD only included stroke and CHD.

### Statistical Analysis

Quantitative variables were presented as median (interquartile range) for the data with non-normal distribution, and the difference between the two groups was compared with the Mann-Whitney *U*-test. Qualitative variables were presented as frequencies and proportions, the difference among groups was compared with Chi-square (χ^2^) test. The dose-response relationships of lipid levels with CVD events and death were characterized by multiple adjusted RCS regression with four knots at the 5th, 35th, 65th, and 95th percentiles. Incidence density was calculated with censor data and Cox proportional hazard regression analysis was performed to estimate the hazard ratios (HRs) and 95% CIs and also multiple factor analysis with adjustment for age, gender, smoking, drinking, BMI, hypertension, diabetes, and lipid-lowering treatments.

For the linear and non-linear relationship analysis of RCS, a *P*-value <0.1 was defined as statistical significance for the non-linearity and linearity tests. A two-tailed *P*-value <0.05 was defined as statistical significance for the association analyses. All statistical analyses were performed with SAS version 9.4 (SAS Institute, Inc, Cary, NC).

## Results

### Baseline Characteristics

Baseline characteristics of 4,128 subjects were summarized in Table 1. During a median duration of 10.75-years follow-up, a total of 272, 189, 434, 519, 157 subjects developed IS, CHD, CVD, all-cause death, and CVD death, respectively. The proportion of subjects with dyslipidemia was 36.07% at baseline. Compared with subjects without dyslipidemia, those with dyslipidemia were more likely to have a higher level of BMI, and higher proportions of hypertension and diabetes (*P* < 0.05). Age, gender, and the proportion of smokers and drinker were comparable between the two groups (Table 1).

**Table 1:** Baseline characteristics of the study population.

**Characteristic**	**All subjects**	**Dyslipidemia**		**Z/χ^**2**^**	P*
		**No**	**Yes**		
Num. of subjects (%)	4,128 (100.00)	2,639 (63.93)	1,489 (36.07)		
Age (year)	59.33 (52.75, 67.07)	59.25 (52.77, 66.78)	59.42 (52.49, 67.75)	0.269	0.788
Gender (%)				1.523	0.217
Male	1,682 (40.75)	1,094 (41.46)	588 (39.49)		
Female	2,446 (59.25)	1,545 (58.54)	901 (60.51)		
Smoker (%)	1,005 (24.35)	667 (25.27)	338 (22.70)	3.427	0.064
Drinker (%)	891 (21.58)	584 (22.13)	307 (20.62)	1.285	0.257
BMI (kg/m^2^)	24.01 (21.93, 26.42)	23.42 (21.37, 25.91)	24.99 (22.76, 27.06)	12.172	<0.001
Hypertension (%)	2,012 (48.74)	1,197 (45.36)	815 (54.73)	33.498	<0.001
Diabetes (%)	465 (11.26)	220 (8.34)	245 (16.45)	62.752	<0.001
TC (mg/dl)	185.33 (162.93, 210.42)	181.85 (162.55, 202.70)	197.68 (164.86, 239.77)	12.760	<0.001
TG (mg/dl)	116.81 (79.65, 176.99)	94.69 (69.91, 131.86)	212.39 (126.99, 283.19)	35.443	<0.001
HDL-C (mg/dl)	51.35 (43.63, 59.85)	53.67 (47.10, 61.39)	43.63 (37.07, 56.76)	21.777	<0.001
LDL-C (mg/dl)	102.32 (84.94, 120.08)	101.16 (86.87, 116.60)	104.63 (81.47, 131.66)	3.976	<0.001
Non-HDL-C (mg/dl)	133.59 (111.68, 157.14)	126.64 (106.95, 146.33)	151.35 (122.39, 183.01)	21.033	<0.001
RC (mg/dl)	29.34 (13.90, 43.63)	23.94 (10.04, 36.68)	40.93 (25.10, 59.85)	22.787	<0.001
ApoAI (g/l)	1.57 (1.38, 1.80)	1.62 (1.43, 1.83)	1.49 (1.30, 1.73)	11.628	<0.001
ApoB (g/l)	0.90 (0.75, 1.08)	0.87 (0.73, 1.02)	0.99 (0.79, 1.17)	12.322	<0.001
Lp(a) (mg/l)	87.75 (43.10, 176.00)	89.1 (44.78, 169.73)	86.75 (40.50, 185.00)	0.986	0.324
ApoB/ApoAI	0.57 (0.46, 0.70)	0.54 (0.44, 0.65)	0.65 (0.52, 0.80)	17.433	<0.001
TC/HDL-C	3.64 (3.07, 4.22)	3.35 (2.87, 3.83)	4.28 (3.69, 4.94)	32.849	<0.001
TG/HDL-C	0.99 (0.63, 1.63)	0.78 (0.53, 1.10)	1.94 (1.25, 2.82)	38.544	<0.001
LDL-C/HDL-C	2.02 (1.64, 2.39)	1.87 (1.53, 2.23)	2.29 (1.91, 2.78)	22.547	<0.001

*Values are presented as median (interquartile range) or n (%).ApoAI, Apolipoprotein AI; ApoB, Apolipoprotein B; BMI, body mass index; HDL-C, high-density lipoprotein cholesterol; LDL-C, low-density lipoprotein cholesterol; Lp(a), Lipoprotein(a); Non-HDL-C, non-high-density lipoprotein cholesterol; RC, remnant cholesterol; TC, total cholesterol; TG, triglycerides. For each quantitative variable, the P-value is obtained by the Mann-Whitney U-test; for each categorical variable, the P-value is obtained through Pearson's χ^2^-test*.

### Dose-Response Relationship Analysis of Lipid Indices and Outcomes

The results of RCS regression analyses are shown in Supplementary Figures 1–13. ApoB had a non-linear association, and TG had a linear association with IS, respectively (*P* < 0.1). TC, LDL-C, ApoAI, and TC/HDL-C ratio had a non-linear association with CHD (*P* < 0.1). Also, ApoB had a non-linear association with CVD (*P* < 0.1). TC had a non-linear association, and non-HDL-C, ApoB, and TC/HDL-C ratio had a linear association with all-cause death, respectively (*P* < 0.1). Moreover, non-HDL-C and RC had a linear association with CVD death (*P* < 0.1).

### Lipid Indices Cut-off Points and Reference Range for Different Outcomes

According to the results of RCS regression analyses, each lipid index was differentially divided into categories by estimating the risk of IS, CHD, CVD, all-cause death, and CVD death. The cut-off points or reference ranges of lipid parameters for different outcome events were presented in Table 2.

**Table 2:** Lipid indices cut-off points and reference range for different outcome events.

**Lipid indices**	**IS**	**CHD**	**CVD**	**All-cause death**	**CVD death**	**Median**	**Clinical reference value**
TC (mg/dl)	185	155–185	155–185	185	185	185	200 (Borderline Elevated), 240 (Elevated)
TG (mg/dl)	115	80–177	115	80–177	115	117	150 (Borderline Elevated), 200 (Elevated)
HDL-C (mg/dl)	40–60	40–60	40–60	40–60	40–60	51	40–60
LDL-C (mg/dl)	105	80–105	80–105	105	105	102	130 (Borderline Elevated), 160 (Elevated)
Non-HDL-C (mg/dl)	105	105–135	105–135	135	135	134	160 (Borderline Elevated), 190 (Elevated)
RC (mg/dl)	30	30	30	30	30	29	–
ApoB (g/l)	0.7–0.9	0.7–0.9	0.7–0.9	0.7–0.9	0.9	0.9	0.8–1.1
ApoAI (g/l)	1.6	1.6–2.1	1.6–2.1	1.6–2.1	1.6–2.1	1.6	1.2–1.6
Lp(a) (mg/l)	90	90	90	90	90	88	200
ApoB/ApoAI	0.4–0.6	0.4–0.6	0.4–0.6	0.6	0.6	0.6	–
TC/HDL-C	5.0	3.6	3.6	3.6	3.6	3.6	–
TG/HDL-C	1.0–3.5	0.6–1.6	1.0–3.5	1.0	1.0	1.0	–
LDL-C/HDL-C	2.0	2.0	2.0	2.0–3.2	1.6–2.4	2.0	–

*ApoAI, Apolipoprotein AI; ApoB, Apolipoprotein B; BMI, body mass index; HDL-C, high-density lipoprotein cholesterol; LDL-C, low-density lipoprotein cholesterol; Lp(a), Lipoprotein(a); Non-HDL-C, non-high-density lipoprotein cholesterol; RC, remnant cholesterol; TC, total cholesterol; and TG, triglycerides; IS, ischemic stroke; CHD, coronary heart disease; CVD, cardiovascular diseases*.

### Association of Lipid and Apolipoprotein With CVD Events and Death

**Figure 2:**
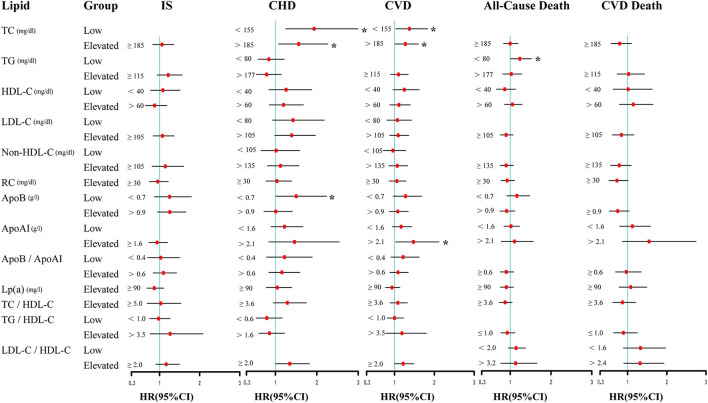
Multivariable adjusted association of lipid indices and IS, CHD, CVD, all-cause death, and CVD death. Cox regression was used to estimate adjusted hazard ratios (HRs) [95% confidence intervals (CIs)] for a low or elevated group of lipid indexes. Adjusted for age, gender, BMI, smoking, drinking, hypertension, diabetes, and lipid-lowering treatment. Lipid indices were divided into two groups (normal group and elevated group, or normal group and low group) or three groups (normal, low, and elevated groups) for different outcomes. Each square has an area inversely proportional to the variance of the log risk. The horizontal lines represent the 95% CI. ApoAI, Apolipoprotein AI; ApoB, Apolipoprotein B; CHD, coronary heart disease; CVD, cardiovascular diseases; HDL-C, high-density lipoprotein cholesterol; IS, ischemic stroke; LDL-C, low-density lipoprotein cholesterol; Lp(a), Lipoprotein(a); Non-HDL-C, non-high-density lipoprotein cholesterol; RC, remnant cholesterol; TC, total cholesterol; TG, triglycerides. *P < 0.05.

The results of Cox proportional hazard regression analysis were summarized in Figure 2. TG ≥ 115 mg/dl (vs. <115 mg/dl) was significantly associated with increased risk of IS (unadjusted HR [95% CI]: 1.329 [1.044–1.691]), while the association was weakened after adjustment for confounding factors (adjusted HR [95% CI]: 1.216 [0.944–1.565]). Compared with the reference interval of 155–185 mg/dl, TC <155 mg/dl and > 185 mg/dl were both significantly associated with the increased risk of CHD (adjusted HRs [95% CIs]: 1.933 [1.248–2.993], 1.561 [1.077–2.261]) and CVD (adjusted HRs [95% CIs]: 1.375 [1.032–1.831], 1.272 [1.01–1.602]). Additionally, ApoAI > 2.1 g/l (vs. 1.6–2.1 g/l) was associated with the increased risk of CVD (adjusted HR [95% CI]: 1.476 [1.031–2.115]) and ApoB <0.7 g/l (vs. 0.7–0.9 g/l) was associated with increased risk of CHD (adjusted HR [95% CI]: 1.502 [1.01–2.234]). Moreover, TG <80 mg/dl (vs. 80–177 mg/dl) was associated with the increased risk of all-cause death (adjusted HR [95% CI]: 1.234 [1.002–1.519]). Furthermore, ApoB ≥0.9 g/l (vs. <0.9 g/l) was associated with decreased risk of CVD death (unadjusted HR [95% CI]:0.702 [0.511–0.964]) while the association was weakened after adjustment for confounding factors (adjusted HRs [95% CIs]:0.749 [0.54–1.038]).

## Discussion

This study systematically analyzed the effects of lipid profiles on CVD events and death. The major findings indicated that lower and higher TC levels were associated with an increased risk of CVD. Lower ApoB levels and higher ApoAI levels were also associated with increased risk of CVD incidence. Furthermore, a lower level of TG was associated with an increased risk of all-cause death.

As an important lipid parameter, TC was used to explore the relationships between cholesterol and CVD events since Framingham Heart Study. The Asia Pacific Cohort Studies Collaboration (APCSC) integrated 30 cohort studies found a positive association between TC and CVD events; each 1 mmol/l increased for TC level was associated with a 41% greater risk of CHD incidence and 23% greater risk of IS incidence (20). However, further subgroup analysis by region indicated that such a relationship was observed among Australia and New Zealand populations but presented a “J” shaped relationship among Asian populations, including Chinese. Additionally, a recent cohort study of 364,486 UK populations free of CVD found a “U” shaped relationship between TC and the risk of CVD incidence (12). The above findings were inconsistent with traditional views but similar to our result, which revealed that lower and higher TC levels were associated with increased risk of CVD incidence. The discrepancy might be explained by the variation of cholesterol levels across countries. The cholesterol level of the Chinese rural population was lower than those in developed countries, in which the proportion of the population with low cholesterol levels was relatively small (21). As a result, it's difficult to find the hazard effect of low TC levels on CVD incidence in those studies carried out in developed countries. Potential mechanisms accounting for the effect of low TC level on CVD may be due to the dysfunction of the cell membrane and the absence in the synthesis of important hormones, including estrogen (22). Estrogens can promote vasodilation, inhibit the renin-angiotensin system (23), reduce blood pressure, and regulate specific inflammatory markers and cytokines (24). Moreover, a multi-ethnic Study of Atherosclerosis of post-menopausal women observed that higher estradiol levels were associated with a lower CHD risk (25).

High-density lipoprotein cholesterol was regarded as “good cholesterol” for its reverse cholesterol transport function, and ApoAI was the important component of HDL for functioning normal biosynthesis (26, 27). Previous studies indicated that the level of ApoAI was inversely associated with CVD events. In the Apolipoprotein Mortality RISk (AMORIS) Study, per 1 SD increase of ApoAI accompanied a 19% reduction of risk of major cardiovascular events (MACE) (28). Similarly, a study from UK Biobank observed that ApoAI per 1 SD (0.27 g/l) increased was inversely associated with risk of CVD, but did not explore potential non-linear relationship (15). In contrast, a significant association of elevated ApoAI with the increased risk of CVD was observed in our study, whereas not for stroke or CHD, the underlying mechanism accounting for the impact of elevated HDL-C and or ApoAI on CVD deserves further exploration.

The key role of LDL-C played in the development and progression of CVD events has been well-illustrated. ApoB was highly related to LDL-C (*r* > 0.9), and one molecule of ApoB was mostly presented in each atherogenic lipoprotein (12, 13, 29). Therefore, ApoB could accurately reflect the total atherogenic lipoprotein in blood and is considered a superior CVD predictor. A cohort from the Stockholm area (Sweden) indicated that elevated ApoB level was a risk predictor of early CVD. Additionally, another large-scale cohort study showed that ApoB was positively correlated with CVD risk; 1 SD increased ApoB, resulting in a 23% increase in CVD risk (12). Unexpectedly, we observed that low ApoB level (< 0.7 g/l) also increased the risk of CHD, and this finding was firstly reported in this study.

In previous studies, TG presented a positive relationship with all-cause death, and elevated TG level was associated with increased all-cause death risk (30, 31). Inconsistent with the above results reduced TG level was associated with a higher risk of all-cause death in our analysis. Recently, some studies proposed the concept of the “TG paradox,” they found that the level of TG showed a negative relationship with the risk of all-cause death in the CVD patients (32, 33). The adverse effects of low TG level could be that TG level was highly associated with BMI (34), low level of TG reflected the poor nutritional status of subjects, and BMI was negatively associated with the risk of all-cause death (35), which named “obesity paradox” (36).

### Strengths and Limitations

The current study has several strengths. First, prospective cohort design spanning over 10 years to investigate the effects of lipid profiles including routine lipids, apolipoproteins, and lipid ratios on the risk of CVD and death, making the conclusion more reliable. Second, the blood lipids levels were comparable to the national level from the 2013–2014 China Chronic Disease and Risk Factor Surveillance (CCDRFS) consisting of 163,641 adults, making the conclusion more representative (37).

There were limitations in this study. Firstly, the sample size is relatively small, which may limit the power of detection. Secondly, we did not collect information on physical activity, which may affect the relationship between lipid and CVD morbidity and mortality. Thirdly, as lipid levels vary among different ethnicities, it could be cautious about generalizing the conclusion to other populations. Thus, a prospective cohort study with a larger sample size and more comprehensive baseline information is warranted to elucidate precise associations between lipid profiles and CVD and death.

## Conclusions

Our findings suggested that the non-linear relationship between TC and CVD events, both lower and higher levels of TC, contributed to the increased risk of CVD. Higher ApoAI levels and lower ApoB levels were associated with the increased risk of CVD, while the lower level of TG was associated with the increased risk of all-cause death. Although the underlying mechanism had not been fully elucidated, maintaining optimal lipid levels would help to prevent CVD and reduce mortality.

## Data Availability Statement

The raw data supporting the conclusions of this article will be made available by the authors, without undue reservation.

## Ethics Statement

The studies involving human participants were reviewed and approved by Nanjing Medical University. The patients/participants provided their written informed consent to participate in this study.

## Author Contributions

JD, SY, and CS: conceived and designed the study. JD, QZ, JS, PW, XZ, YC, XC, ML, LW, CC, YF, and CS: data collection, analysis, and interpretation. JD: drafted the manuscript. XC, YF, and CS: revised the manuscript critically for intellectual content. All authors contributed to the article and approved the submitted version.

## Funding

This work was supported by grants from the National Natural Science Foundation of China (Grant Nos. 81872686 and 81573232), Jiangsu Provincial Fourth 333 Project, the Priority Academic Program Development of Jiangsu Higher Education Institutions (Public Health and Preventive Medicine), and the Flagship Major Development of Jiangsu Higher Education Institutions. The Funders had no role in the design and conduct of the study, collection, management, analysis, and interpretation of the data, preparation, review, or approval of the manuscript, and decision to submit the manuscript for publication.

## Conflict of Interest

The authors declare that the research was conducted in the absence of any commercial or financial relationships that could be construed as a potential conflict of interest.

## Publisher's Note

All claims expressed in this article are solely those of the authors and do not necessarily represent those of their affiliated organizations, or those of the publisher, the editors and the reviewers. Any product that may be evaluated in this article, or claim that may be made by its manufacturer, is not guaranteed or endorsed by the publisher.
