# Supplementary material for: The Associations of Lipid Profiles With Cardiovascular Diseases and Death in a 10-Year Prospective Cohort Study
Source: Front Cardiovasc Med. 2021 Nov 25;8:745539. doi: 10.3389/fcvm.2021.745539 (PMC8655628; doi:10.3389/fcvm.2021.745539)
Supplement: Supplementary file 1 [file Data_Sheet_1.PDF]

Supplementary Figures

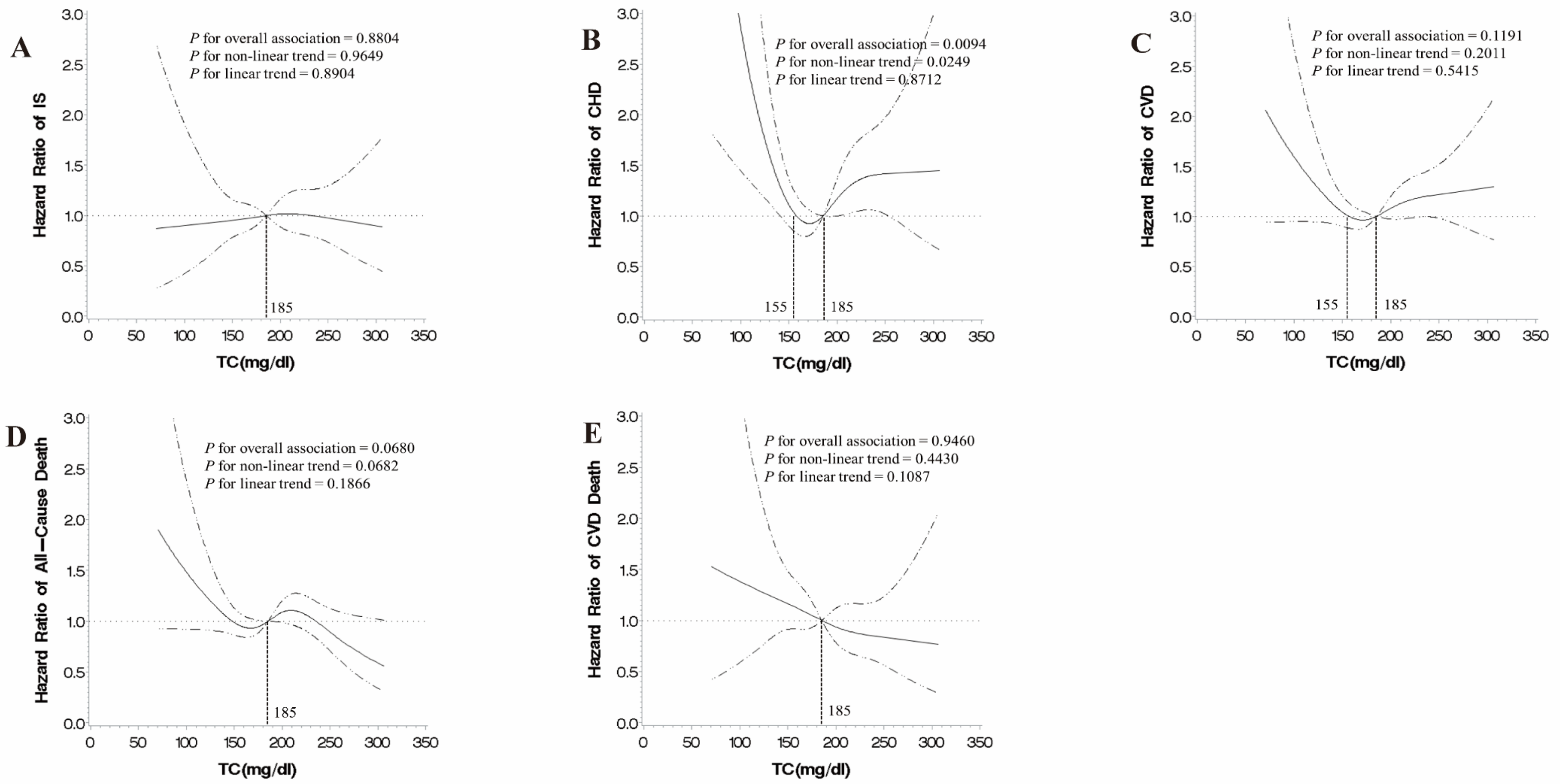

Supplementary figure 1. Restricted cubic spline cox regression analysis of TC and the risk of IS, CHD, CVD, All-Cause death, and CVD Death. Abbreviation: TC: total cholesterol; IS: ischemic stroke; CHD: coronary heart disease; CVD: cardiovascular diseases.

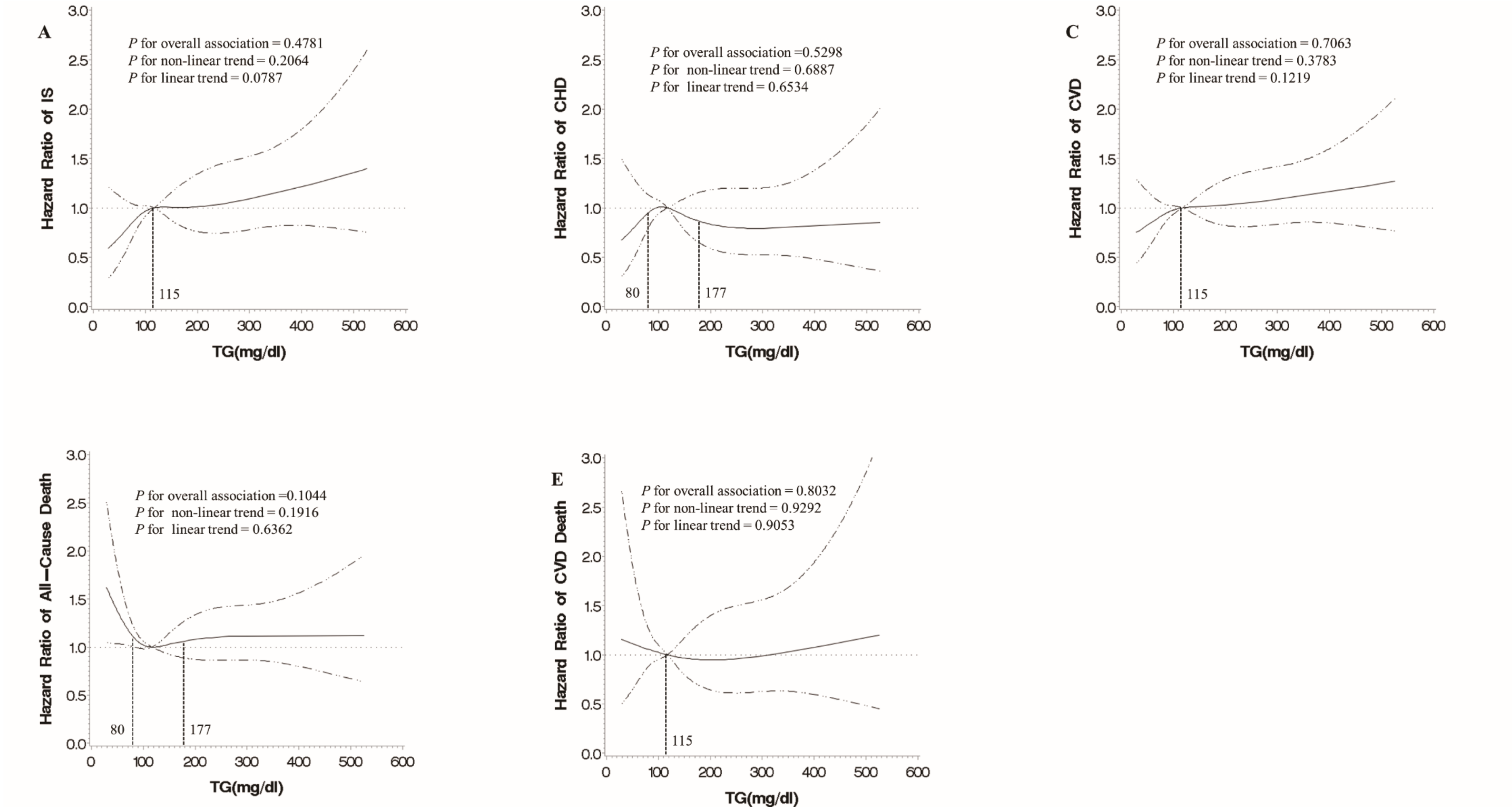

Supplementary figure 2. Restricted cubic spline cox regression analysis of TG and the risk of IS, CHD, CVD, All-Cause death, and CVD Death. Abbreviation: TG: triglycerides; IS: ischemic stroke; CHD: coronary heart disease; CVD: cardiovascular diseases.

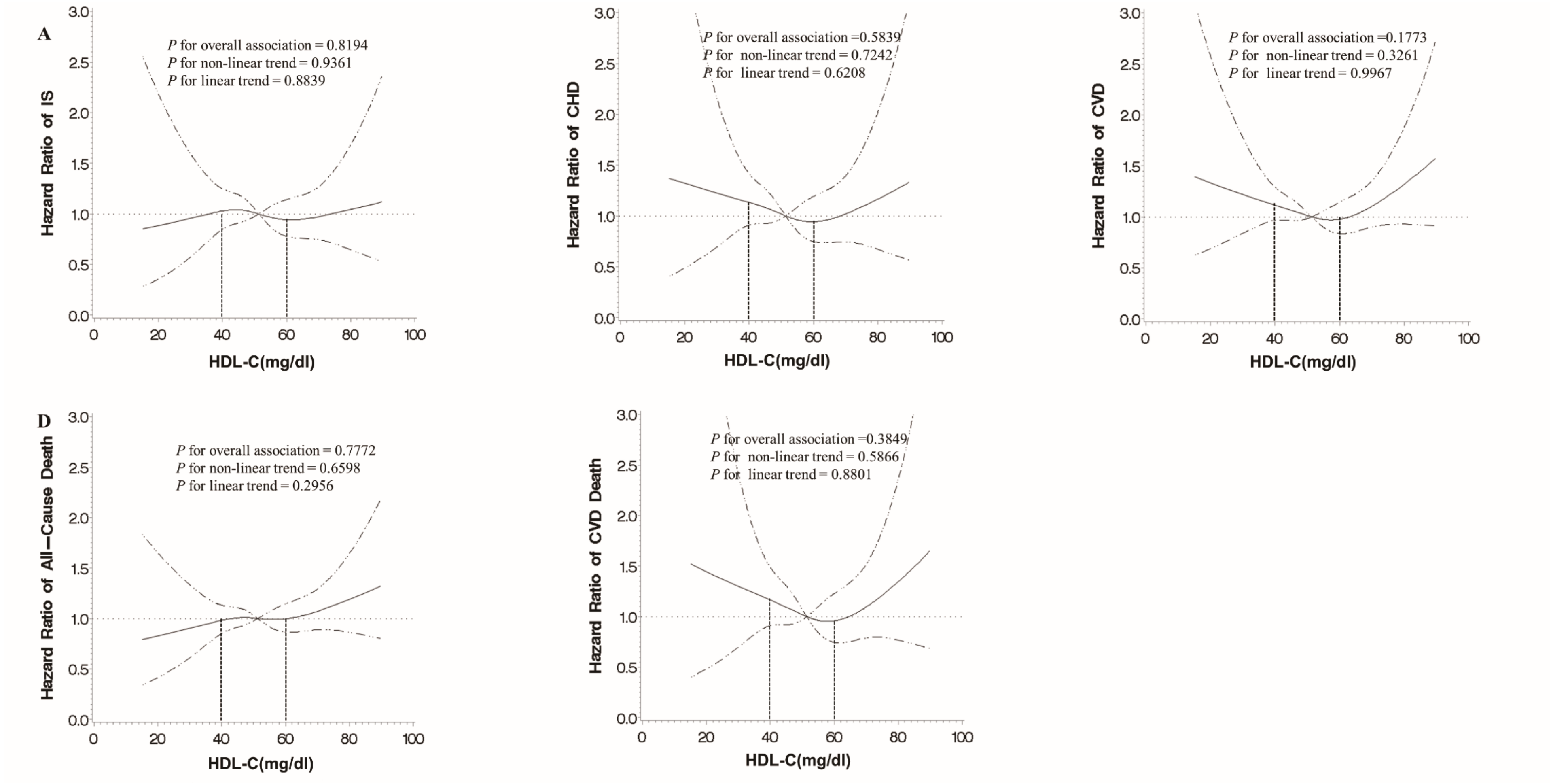

Supplementary figure 3. Restricted cubic spline cox regression analysis of HDL-C and the risk of IS, CHD, CVD, All-Cause death, and CVD Death. Abbreviation: HDL-C: high-density lipoprotein cholesterol; IS: ischemic stroke; CHD: coronary heart disease; CVD: cardiovascular diseases.

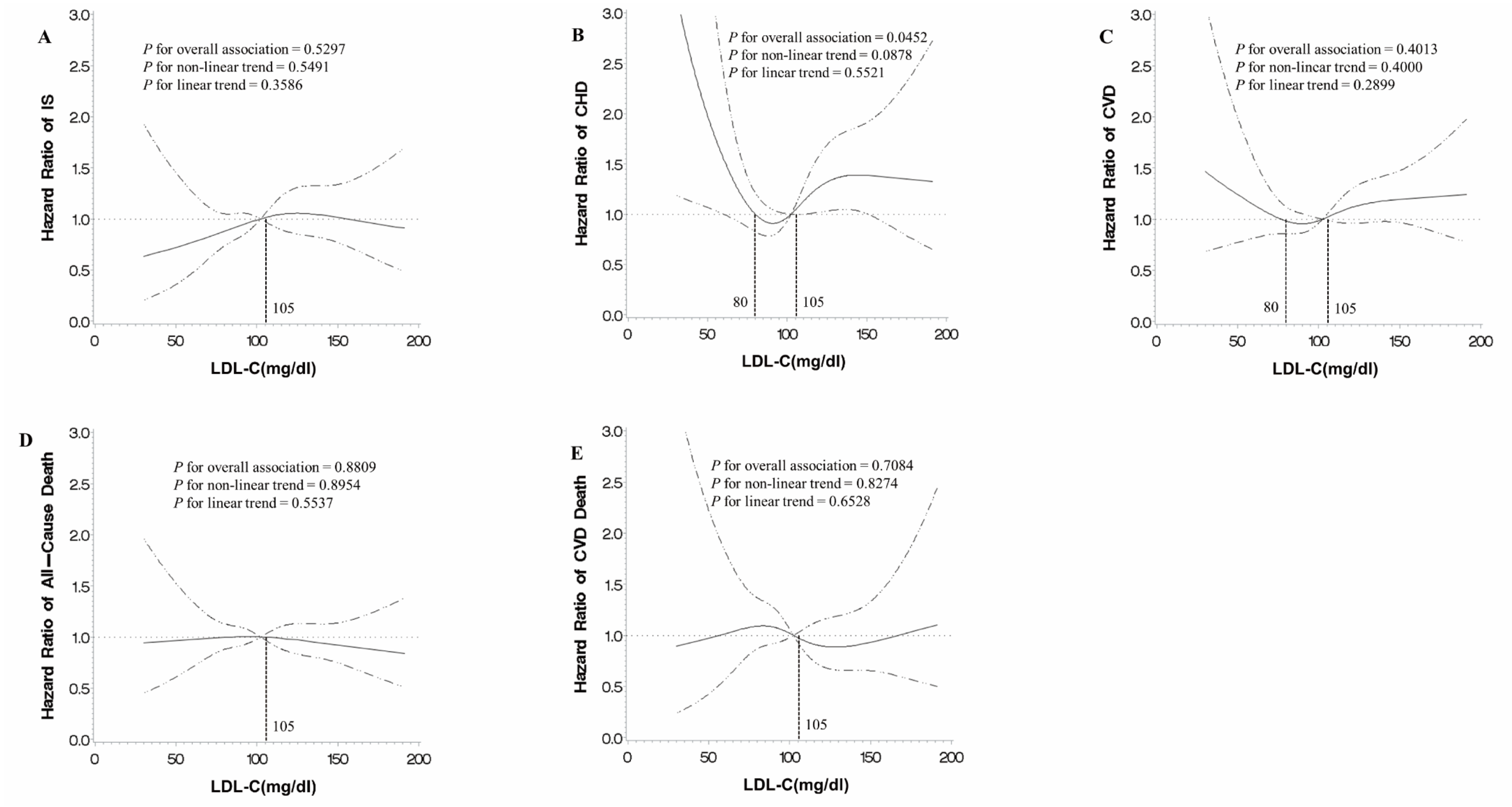

Supplementary figure 4. Restricted cubic spline cox regression analysis of LDL-C and the risk of IS, CHD, CVD, All-Cause death, and CVD Death. Abbreviation: LDL-C: low-density lipoprotein cholesterol; IS: ischemic stroke; CHD: coronary heart disease; CVD: cardiovascular diseases.

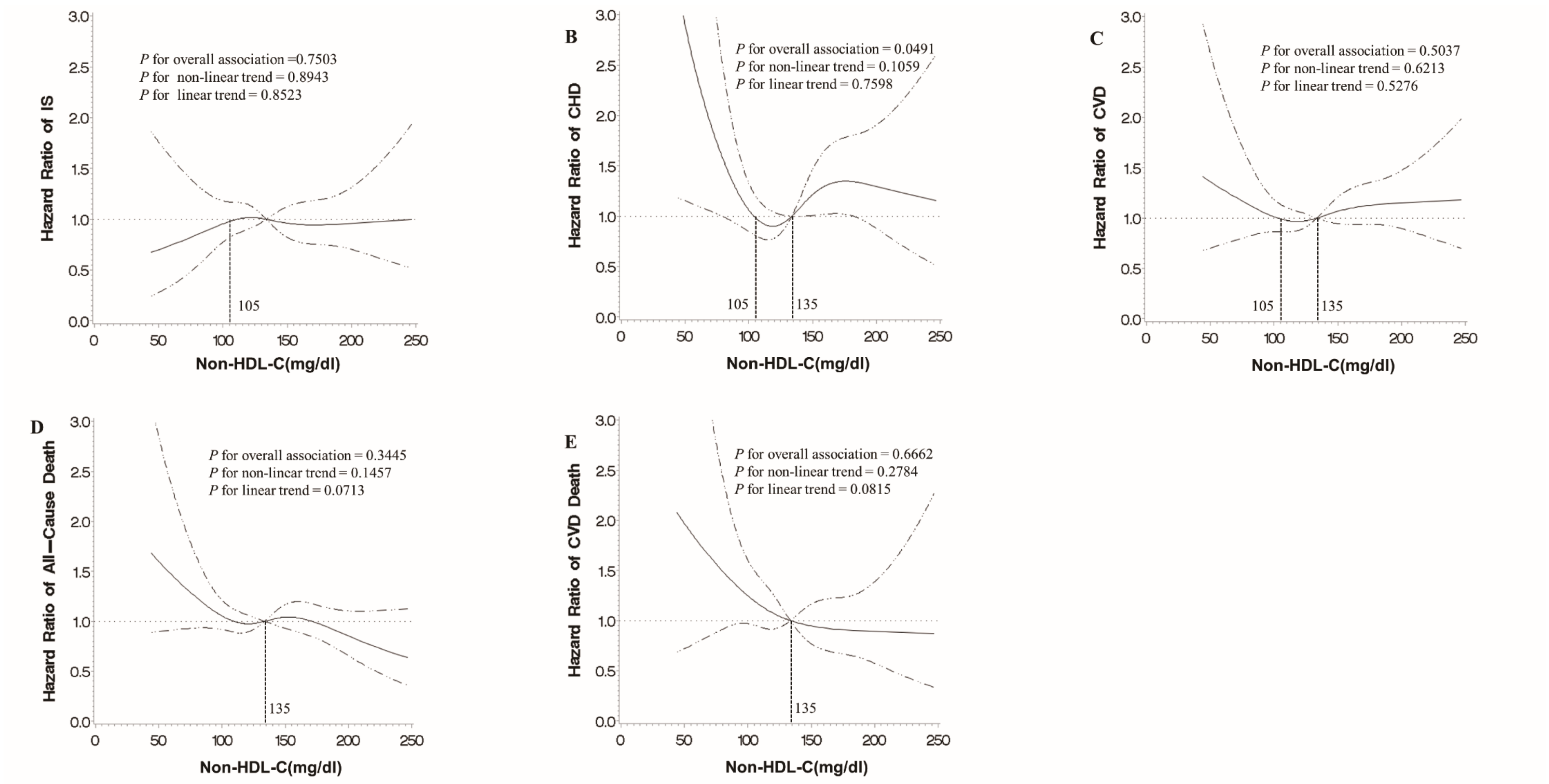

Supplementary figure 5. Restricted cubic spline cox regression analysis of Non-HDL-C and the risk of IS, CHD, CVD, All-Cause death, and CVD Death. Abbreviation: HDL-C: non-high-density lipoprotein cholesterol; IS: ischemic stroke; CHD: coronary heart disease; CVD: cardiovascular diseases.

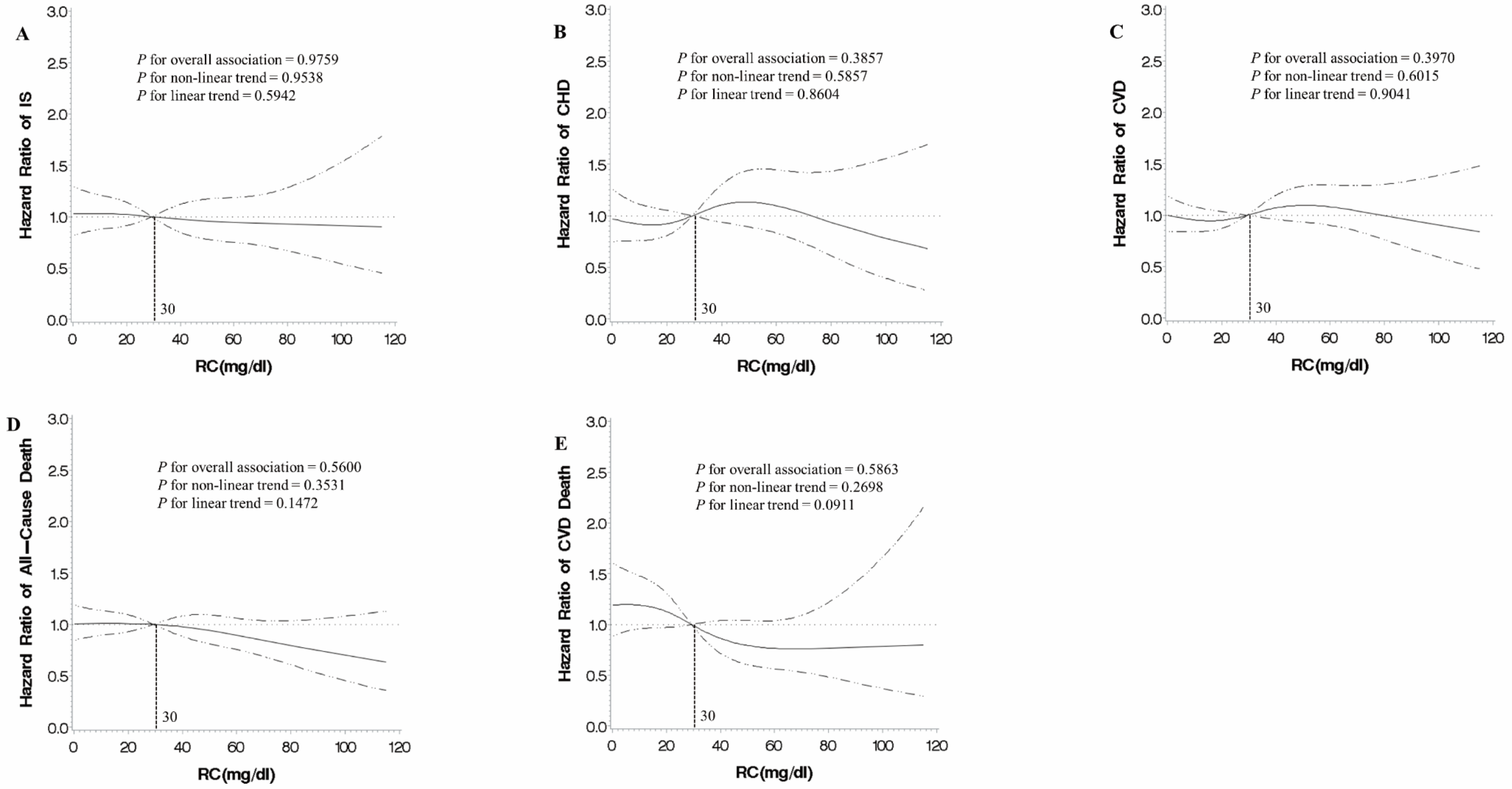

Supplementary figure 6. Restricted cubic spline cox regression analysis of RC and the risk of IS, CHD, CVD, All-Cause death, and CVD Death. Abbreviation: RC: remnant cholesterol; IS: ischemic stroke; CHD: coronary heart disease; CVD: cardiovascular diseases.

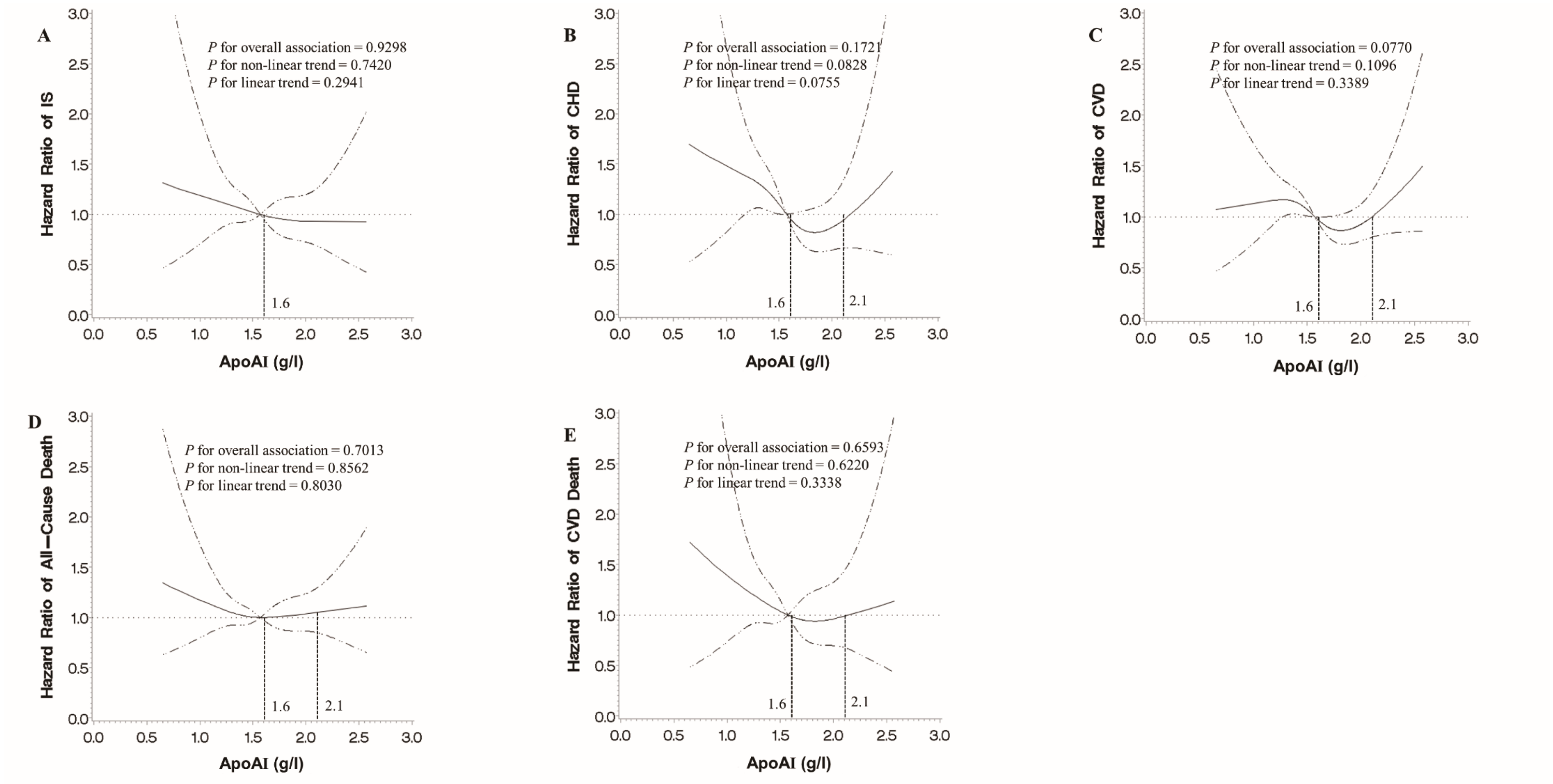

Supplementary figure 7. Restricted cubic spline cox regression analysis of ApoAI and the risk of IS, CHD, CVD, All-Cause death, and CVD Death.
Abbreviation: ApoAI: Apolipoprotein AI; IS: ischemic stroke; CHD: coronary heart disease; CVD: cardiovascular diseases.

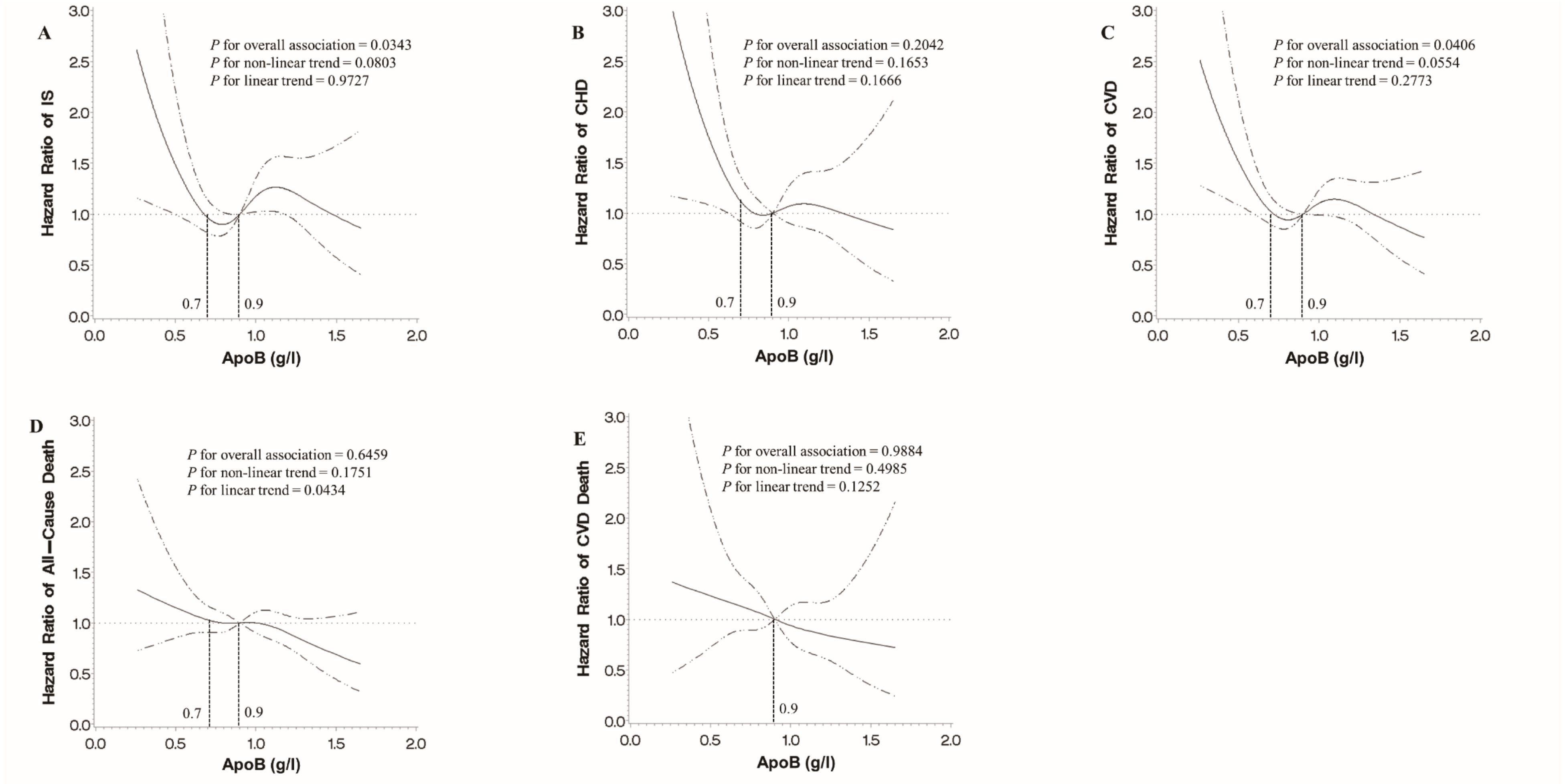

Supplementary figure 8. Restricted cubic spline cox regression analysis of ApoB and the risk of IS, CHD, CVD, All-Cause death, and CVD Death. Abbreviation: ApoB: Apolipoprotein B; IS: ischemic stroke; CHD: coronary heart disease; CVD: cardiovascular diseases.

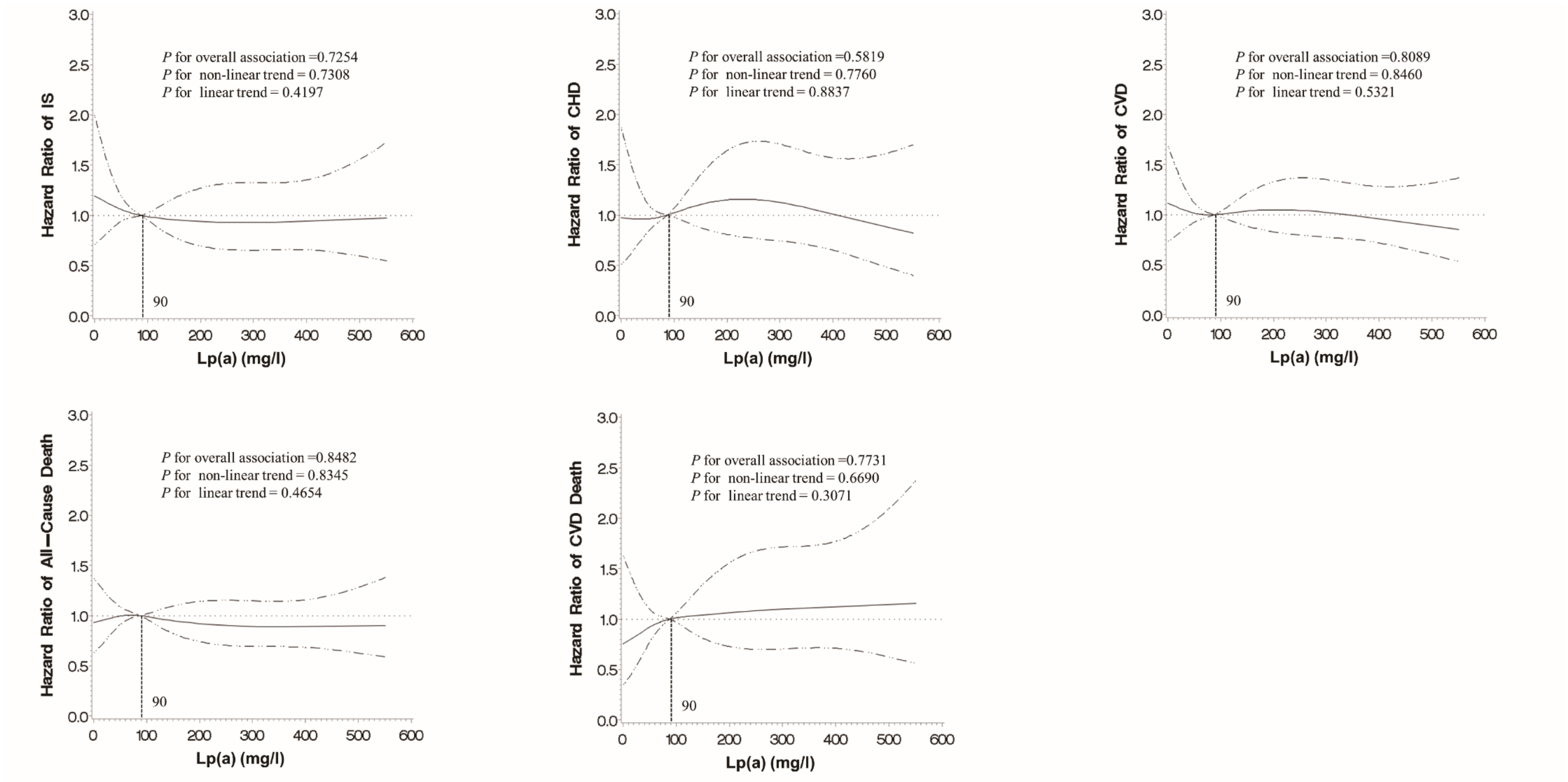

Supplementary figure 9. Restricted cubic spline cox regression analysis of Lp(a) and the risk of IS, CHD, CVD, All-Cause death, and CVD Death. Abbreviation: Lp(a): Lipoprotein(a); IS: ischemic stroke; CHD: coronary heart disease; CVD: cardiovascular diseases.

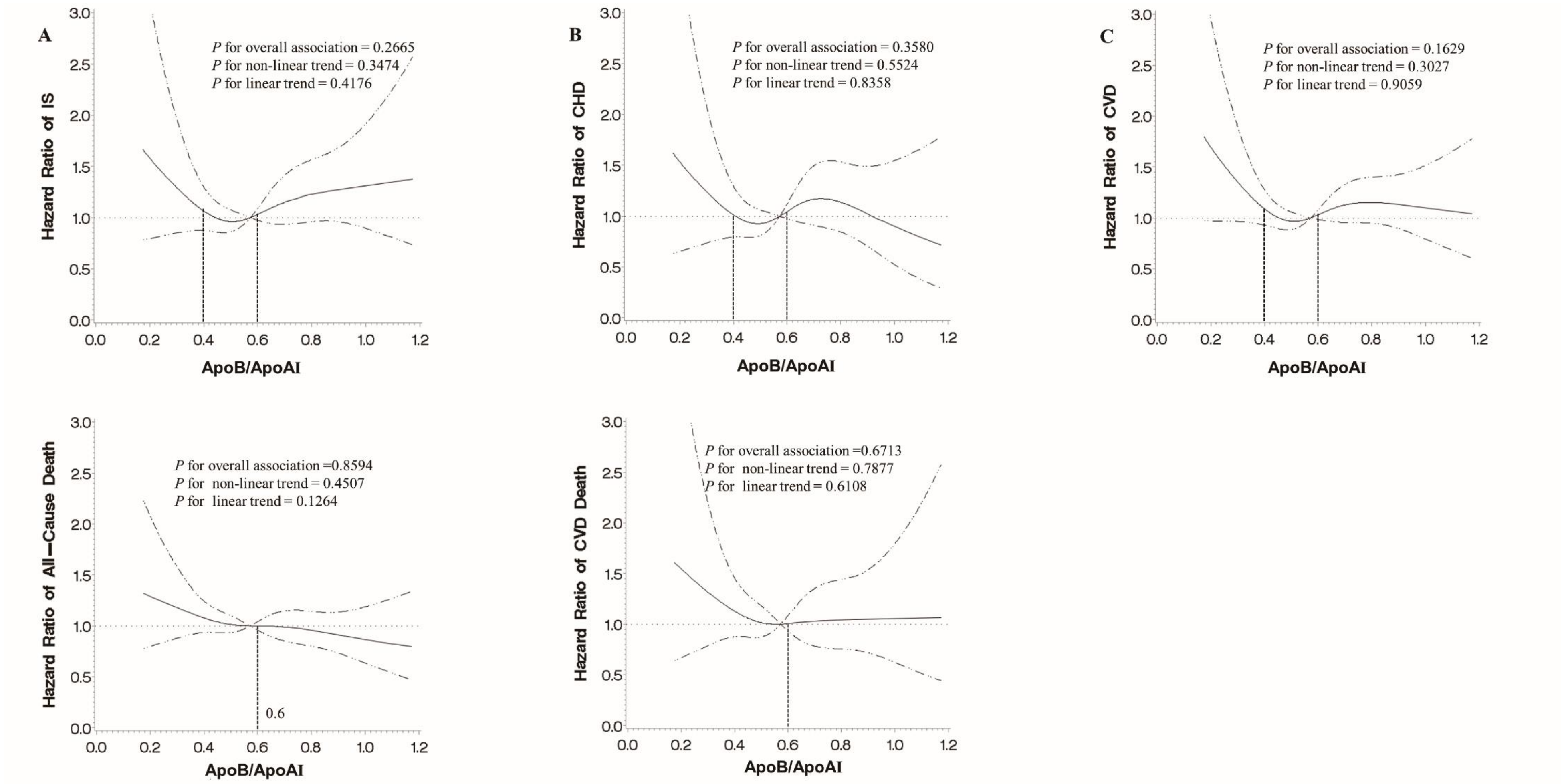

Supplementary figure 10. Restricted cubic spline cox regression analysis of ApoB/ApoAI and the risk of IS, CHD, CVD, All-Cause death, and CVD Death.

Abbreviation: ApoAI: Apolipoprotein AI; ApoB: Apolipoprotein B; IS: ischemic stroke; CHD: coronary heart disease; CVD: cardiovascular diseases.

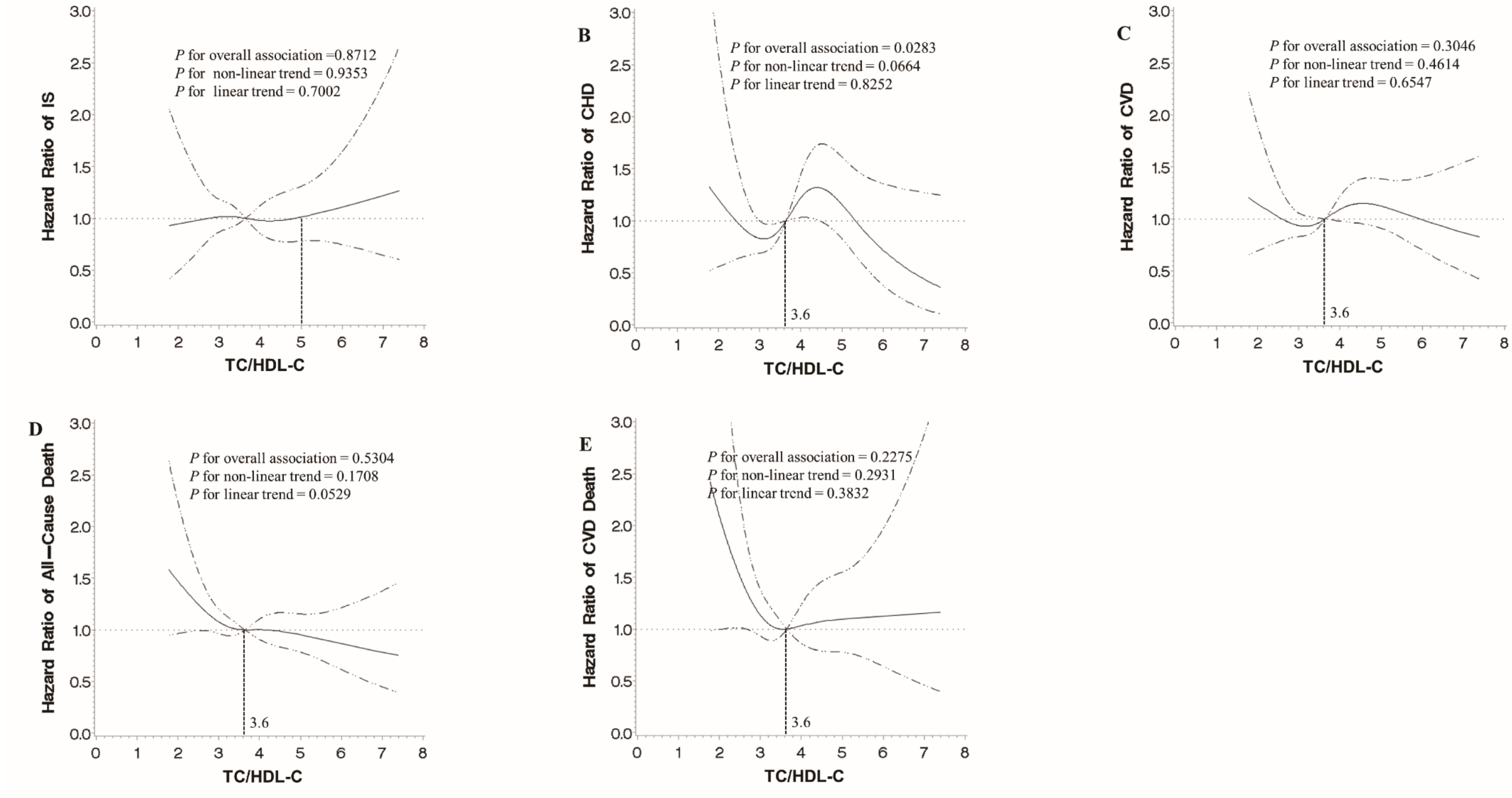

Supplementary figure 11. Restricted cubic spline cox regression analysis of TC/HDL-C and the risk of IS, CHD, CVD, All-Cause death, and CVD Death. Abbreviation: HDL-C: high-density lipoprotein cholesterol; TC: total cholesterol; IS: ischemic stroke; CHD: coronary heart disease; CVD: cardiovascular diseases.

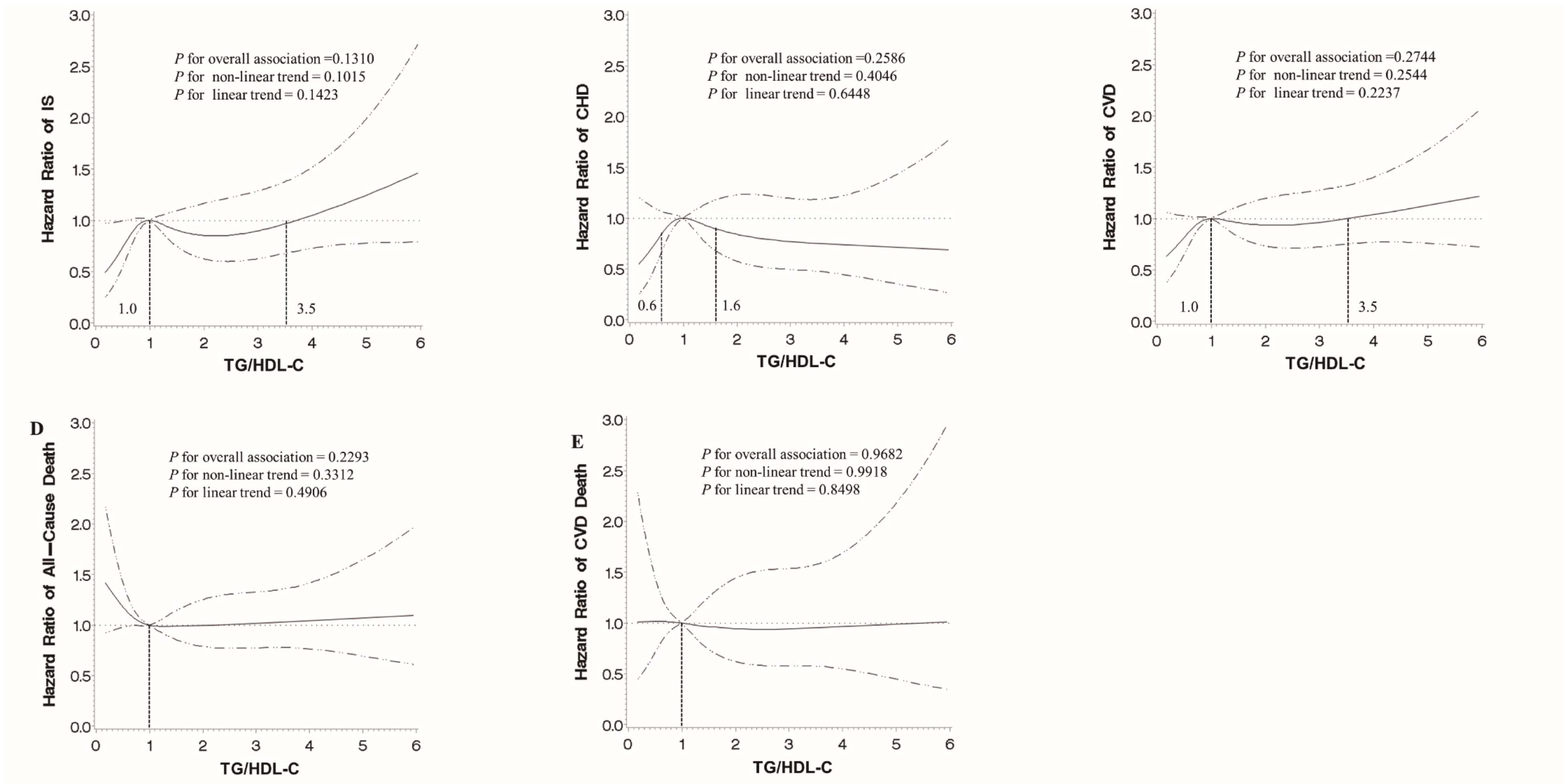

Supplementary figure 12. Restricted cubic spline cox regression analysis of TG/HDL-C and the risk of IS, CHD, CVD, All-Cause death, and CVD Death. Abbreviation: HDL-C: high-density lipoprotein cholesterol; TG: triglycerides; IS: ischemic stroke; CHD: coronary heart disease; CVD: cardiovascular diseases.

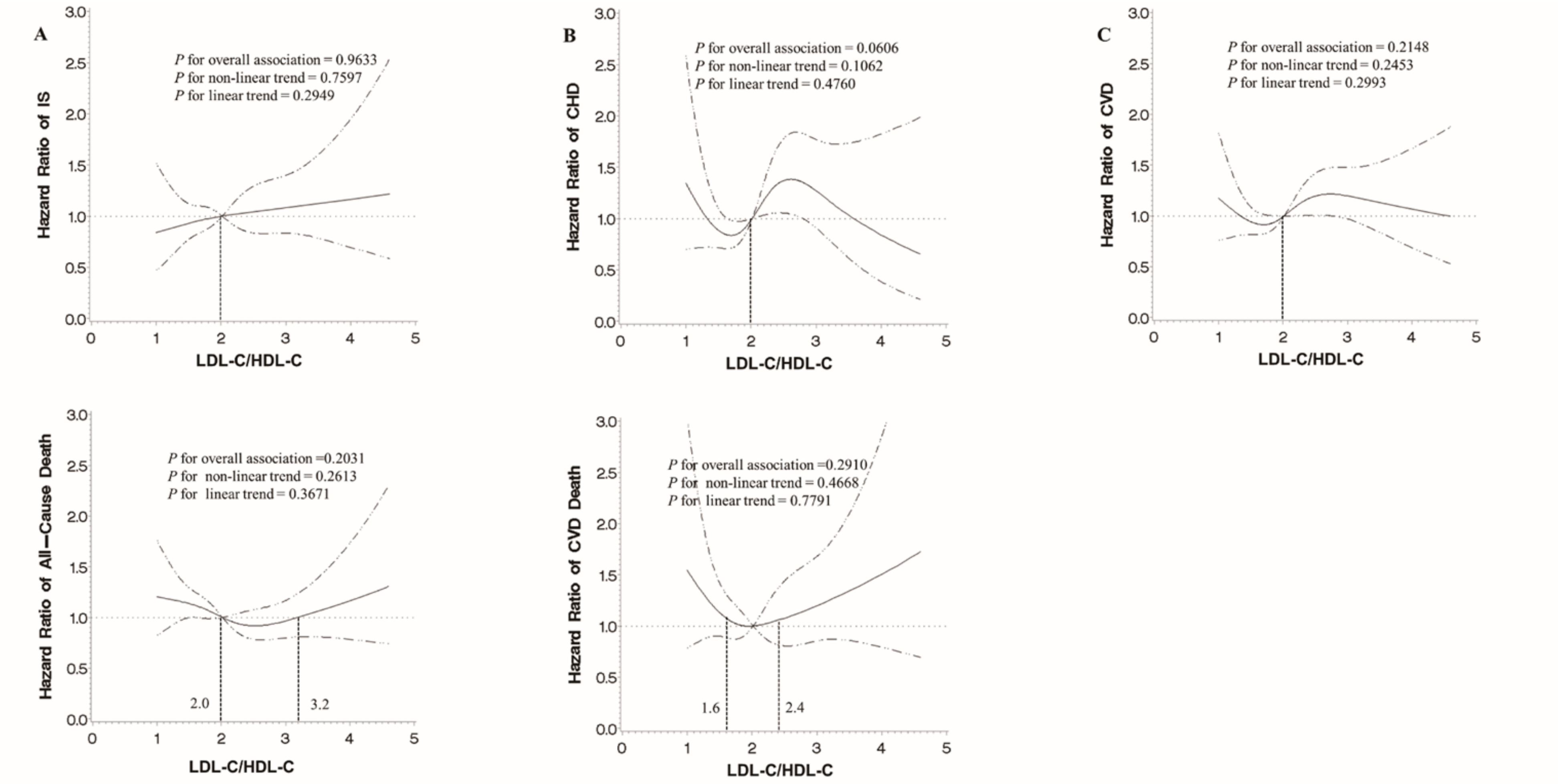

Supplementary figure 13. Restricted cubic spline cox regression analysis of LDL-C/HDL-C and the risk of IS, CHD, CVD, All-Cause death, and CVD Death.

Abbreviation: HDL-C: high-density lipoprotein cholesterol; LDL-C: low-density lipoprotein cholesterol; IS: ischemic stroke; CHD: coronary heart disease; CVD: cardiovascular diseases.
